# Implications of COVID-19 labour market shock for child and household hungers in South Africa: Do social protection programs protect?’

**DOI:** 10.1371/journal.pone.0269848

**Published:** 2022-07-01

**Authors:** Dambala Gelo, Johane Dikgang

**Affiliations:** 1 School of Economics and Finance, University of the Witwatersrand, Johannesburg, South Africa; 2 Department of Economics and Finance, Florida Gulf Coast University, Fort Myers, Florida, United States of America; 3 The Water School, Florida Gulf Coast University, Fort Myers, Florida, United States of America; University of Jyvaskyla, FINLAND

## Abstract

**Background:**

Recent studies have confirmed that the COVID-19 lockdown has caused massive job losses. However, the impact of this loss on food security is not well-understood. Moreover, a paucity of evidence exists regarding social protection grants’ countervailing effects against such shocks. This study examined the effects of job loss (labour income loss) on child and household hungers (our two measures food insecurity) during COVID-19 pandemic in South Africa. It also ascertained whether these effect were offset by alternative social grant programs to document the protective role of the latter.

**Data and methods:**

We used South Africa’s National Income Dynamics Study (NIDS) and the Coronavirus Rapid Mobile Survey (CRAM) data. These data cover a nationally representative sample of 7073 individuals. We employed a probit model to estimate the effect of job loss and receipts of various social grants on child and households’ hungers. We also estimated the double-selection logit model to account for the model’s uncertainty surrounding the variable selection and treatment-effects estimation using lasso (Telasso) for causal inference of our analysis.

**Results:**

Our analyses showed that households exposed to a labour market shock during the pandemic experienced a significant increase in our measures of food insecurity (child and household hungers). Specifically, we found that compared with households containing employed respondents, households with respondents who lost their jobs due to COVID-19 lockdown were 5.4% more likely to report child hunger and 2.6% more likely to report household hunger in the past seven days A receipt of child support grant reduces the likelihood of reporting child hunger and household hunger by 21.7%and 16.9% respectively among these households. A receipt of old age pension grant reduces the likelihood of reporting household hunger by 24% with no significant effect on child hunger.

**Conclusion:**

The COVID-19 lockdown resulted in unprecedent job losses with significant implications for food insecurity. Job loss due to COVID-19 lockdown significantly increased food insecurity in South Africa. Receipts of social grants effectively offset this adverse effect. The protective effect of the social grant is heterogenous across its alternative programs (child support grant and old age pension grant) and food insecurity, suggesting the differences in the size of transfers and motivations for sending these transfers.

## 1. Introduction

In March 2020 the World Health Organization (WHO) declared the outbreak of the COVID-19 pandemic, which led governments around the world to take unprecedented responses to contain the spread of the virus. In response to the WHO, governments implemented lockdown policies of various degrees. Inevitably, these measures represented a shock for worldwide economy, owing to a sharp reduction in almost all economic activities. More specifically, the lockdown caused lack of employment, production, and demand in numerous industries, which in turn summed up into a macroeconomic downturn [1].

Specifically, COVID-19 lockdown increased food insecurity in two major ways [2, 3]. First, the lockdown disrupted agricultural and food markets through labour shortages imposed by restrictions on people’s possibility to travel. In addition, export restrictions imposed by certain countries also disrupted trade flows for staple foods, such as wheat and rice. Combined, these restrictions led to hikes in food prices [4, 5]. This raised concern that poverty and food insecurity would rise, and that the nutritional status of low-income households and vulnerable groups would possibly fall [4].

Second, the lockdown restriction spurred food insecurity by causing loss of income and remittances. COVID-19 lockdown restrictions resulted in increased unemployment in various countries worldwide [6]. From a consumer perspective, the resultant reduction in income undermines purchasing power for food and other consumption goods and services particularly among the vulnerable [7]. United Nations World Food Program (WFP) forecasts that the rate of acute food insecurity will double globally by the end of 2020 due to income and remittance losses, and disruption of food systems following the pandemic shock [8, 9].

Following the COVID-19 lockdown, a few studies have investigated the food insecurity consequences of the restrictions in different countries. A panel data study based on representative household survey confirmed that the lockdown did not affect food consumption and household dietary diversity in Ethiopia [7]. However, another panel data study of a difference-in-difference (DID) analysis reported that the lockdown led to a 6%-15% increase in experience of food insecurity in Nigerian households [10]. Similarly, a cross-sectional data study found that the lockdown worsened food insecurity in Kenya and Uganda, with farm households being likely to be less affected compared to those who completely depended on market sources for food [11]. However, none of these studies ascertained the specific pathway (the mechanism) through which the restrictions impacted food insecurity and other welfare outcomes. More specifically, current studies fall short of unpacking the specific effect of food price rise or job loss (labour income shock) or both on food security during lockdown.

Moreover, as the level of lockdown restrictions and their implementation vary across countries, so do their welfare effects. Even within a country, the lockdown’s food insecurity effects vary across geography (urban versus rural), livelihood options, and pre-existing vulnerabilities to food insecurity [2, 12, 13]. Such potential heterogeneity of the lockdown’s food-insecurity effects raises concerns about the generalizability of previous studies’ results. Thus, uncertainties around the external validity of previous evidence on the consequences of lockdown on food insecurity warrant context-specific studies that account for a specific pathway through which such effects emerge. Furthermore, although unemployment is found to be a key determinant of poverty [14], there is a paucity of evidence on the effect of job loss shock on household food insecurity [15, 16] and other welfare outcomes in South Africa [14].

Our study sets out to investigates the causal effects of labour income loss on food insecurity during the coronavirus pandemic in South Africa. It also documents the protective role of social grant (income transfer) programs against food insecurity effect of the labour income shock. In effect, our analysis contributes to emerging empirical evidence on food insecurity/welfare effects of the pandemic and the related lockdown policies.

South Africa adopted COVID-19 lockdown on March 15 and went into a total lockdown on March 26 –designated Level 5 restrictions allowing only essential travel and services. The resultant drop in economic activities led to an involuntary workforce displacement with significant implication for labour income loss, especially among the vulnerable workers. Recent estimates show that between 2.2 and 2.8 million adults in South Africa lost jobs within a period of February to April 2020 following the lockdown [17]. The job loss in turn caused an enormous reduction in labour income among households largely dependent on labour. Approximately 1 in 3 of those who earned income in February 2020 did not earn an income in April 2020 due to job losses [18].

South Africa is an interesting case study for the following major reasons. First, millions of its people were already living below the national poverty line and inequality is the highest in the world [19]. Inequality within labour markets resulting from–rising unemployment and rising labour income inequality is a major cause driving the growing level of aggregate inequality in South Africa before the outbreak of the pandemic [20]. As the lockdown induced job losses were disproportionately concentrated among already disadvantaged groups in the labour market [21], it follows that the South Africa lockdown is likely to exacerbate this existing inequality.

Second, the reinforced inequality in labour income is likely to exacerbate inequalities in food security. To be specific, a fall in labour income, inter alia, undermines food security and this effect is likely to be disproportionately concentrated among already disadvantaged groups in the labour market depending on household or individual access to social protection and/or market insurance e.g., the unemployment insurance fund (UIF). In 2018 (pre-lockdown time), it was estimated that 21% of all households ran out of money to buy food in the past 12 months whereas 10% of households with no income from grants or unemployed reported skipping meals for 5 days out of the past 30 days [22]. However, it is not clear whether these situations have been worsened due to lockdown.

Note though that the effects of income shock on food insecurity depend on the insurance/protection of total income against job loss either through households’ income smoothing (diversified income portfolio), or social protection/a safety net. In the South African context, in essence, income for poorer households is expected to be significantly insulated against job loss by social protection (government transfer) [1]. Nevertheless, either job loss or a business shutdown, or both, represented a significant shock among a considerable proportion of grant recipient households compared to pre-lockdown times. These sources represented significant portions of total income among most of these households [16]. It was reported that 75% of grant receiving households were earning income from sources such as from employment, business, or remittances in addition to a grant receipt, while 42% of them lost the non-grant main source of household income following the lockdown.

In fact, the occurrence of a main income source loss among both grant receiving and non-grant receiving households suggests a varying food insecurity effect and other welfare costs of COVID-19 lockdown restriction across these households’ categories. Against this backdrop, the present study also ascertained the heterogeneity of the food insecurity effect of lockdown-induced unemployment.

Overall, unpacking the causal effects of job loss/labour income loss and the receipt of social grant on food insecurity during the coronavirus pandemic provides a range of useful policy insights. More importantly, it helps identify vulnerable populations in the pandemic, informs targeting policy interventions, and shed light on sufficiency of the current income transfer policies in protecting those vulnerable to labour market shocks and their food insecurity implications. We used South African nationally representative data to estimate the effect of job loss/labour income loss and social protection on outcomes related to food insecurity. Building on recent literature that estimated the causal effect of involuntary job loss on health outcomes [11, 12], we exploited exogenous variation in unemployment to identify the causal effects of job loss on food insecurity. As lockdown restrictions exogenously determine business/firm closure, the source of unemployment is beyond the control of individuals [23, 24]. This exogenous variation in unemployment, conditional on a range of observable individual and labour market characteristics, allowed us to provide causal estimates of the effects of job loss on food insecurity.

## 2. Materials and methods

### 2.1 Data

In this study, we used unique longitudinal data collected from two waves of South Africa’s National Income Dynamics-Coronavirus Rapid Mobile Survey (NIDS-CRAM). Respondents were selected from a nationally representative stratified sample, and information was collected using computer-assisted telephone interviews.

The sampling design followed two-stage stratified cluster sampling but with "batch" sampling. The representative sample was obtained by drawing the NIDS-CRAM survey participants from the already existing National Household Survey of the National Income Dynamics Study (NIDS) wave 5. The NIDS-CRAM sample was obtained from the 2017 National Sample of the NIDS. Batch sampling implies that sampled individuals were sent to the fieldwork team in batches of 2500 individuals. The individuals are randomly drawn within each of the 99 strata, which are a combination of household per capita income decile, race, age and urban/rural. As information about the response rates from the initial batches of 2500 respondents each was obtained, the batch sampling method enabled for changes in the number of individuals sampled in each stratum in the subsequent batches. More individuals from the strata with lower response rates were sampled, and fewer individuals were sampled from strata with higher response rates. This process continued until the target number of successfully interviewed respondents in each stratum was achieved, or the individuals in the stratum have been exhausted. The initial survey (wave 1) distributed 17 568 questionnaires, 7 074 were returned [25], and 7 073 provided useable information. The data for this paper comes from the two waves of the NIDS-CRAM panel. The first wave of this data was collected between May 7^th^ and June 27^th^, 2020 (during stages 3 and 4 of the national lockdown).

#### Food insecurity variables

Following [10, 23, 26, 27], we measure food insecurity using two indicators: experience of child hunger and household food shortage in the last 7 days. To be specific, our first indicator captures whether a child went hungry due to not having adequate food in the past 7 days. The second indicator measures instances where a household run out of money to buy food in the past 7 days.

Ideally/clinically speaking, food insecurity would be assessed using the multidimensional indicator of 9-items [28]. Despite not capturing all dimensions of food insecurity, our food insecurity indicators certainly represent food consumption shortfall (dietary outcome) and hence are of interest on own right to ascertain welfare consequence of income shocks like the one due to labour market shock following the COVID-19 pandemic breakout [10, 23, 26]. Our second indicator, food insufficiency measured in terms of inadequate amount of food intake due to a lack of money or resources, has been shown to be a valuable proxy of food insecurity. In fact, it is important to note that consumption is an input into the formation and maintenance of human capital, implying that its shortfall not only have short-run welfare loss (decreased individual utility due to consumption fall), but may also have long-term consequences that undermine future productivity/income [29]. Overall, considering these implications, the use of these indicators, as measures of consumption shortfalls, to investigate the welfare implications of shocks is readily justified in the absence of 9-item food security index/scale.

*Job loss*. For the empirical analysis, we considered a sample comprising all the adults who were employed in the month before the COVID-19 lockdown came into effect. From the first wave of the NIDS-CRAM survey, we obtained retrospective information on whether adults had been working in February 2020, prior to the imposition of South Africa’s first lockdown. Note that wave 1 survey asked respondents about employment status in February and April 2020. This is important because the lockdown started on March 26, which implies that February represents the pre-pandemic period while April represents the pandemic period.

During the first lockdown, which was referred to as alert level 5, all non-essential economic activities were suspended. Moreover, the first wave of the NIDS-CRAM survey collected detailed current data, inter alia, on income, demographic variables, receipt of social grants, and knowledge and behaviour related to COVID-19.

Following [24], we determined job loss due to lockdown by identifying the permanent layoff between February and April 2020. To be specific among respondent who retrospectively reported to be working in February 2020, we identified the following categories; (i) adult who were still working and earning a non-zero income in April 2020, (ii), adult who were not working but on paid leave, (iii) adult who were furrowed (neither working nor earning an income but had a job to return to, and (iv) adults who permanently lost job (laid off) following the lockdown.

### 2.2 Empirical strategies

Our outcome variables of food insecurity rate of child and household hungers represent a binary variable, respectively. This suggests the effective employment of a logit model to estimate the effect of job loss and receipt of various social grants on household and child food insecurity during COVID-19. As mentioned in the previous section, our results provide causal estimates of these effects given that the COVID-19 pandemic and the consequent national lockdown caused exogenous job loss. Moreover, we claimed that estimates of the effects of social grants are causal, as the rollout of each involves natural experiments.

As a baseline, we started our analysis by estimating the traditional logit model. However, while one can perform a series of specification tests to select among alternative models that better fit the data, there is no guarantee that the results of these tests would help us select the controls that are most relevant. To overcome such model uncertainty, we applied the regularized linear regression method to machine learning.

Specifically, drawing on recent advances in statistics and econometrics, we used a two-step method of least absolute shrinkage and selection (lasso) regression of logit model following, [30–32] as a practical solution to the problem of variable/model selection. We applied these approaches to Eq (1) below in its logit or logit specification.

$$Y_{i}=\beta_{0}{+\beta }_{1}T_{i}+\beta_{2}G_{i}+\beta_{2}T_{i}*G_{i}+\beta_{3}X_{i}+\epsilon_{i}$$ (1)

where *Y_i_* is a binary or count variable of food insecurity outcomes, *T_i_* is a dummy that takes a value of one, if an individual *i* lost a job or furloughed, *G_i_* is dummy that takes value of 1 if an individual receives social grant, and *X_i_* is a vector of control covariates. Exogenous variation in *T_i_*, and due to their corresponding natural experiments, means that neither of them is correlated to the error term *ϵ_i_* suggesting that estimation of (1) yields estimates of parameters, *β*_1_ measuring the average impacts of job loss following due to COVDI-19 lockdown and *β*_2_ measuring the average effect of social grant receipt by a household among households with a member that lost a job.

Note though that lasso regression estimates of job loss may not be amenable to causal interpretation as experience of labour market shock is likely to be conditional on observable individual and labour market characteristics. However, this concern doesn’t apply to social grant receipts as they are based on exogenously given eligibility criterion. In the interest of causal inference of the effect of labour market shock on food insecurity, we employed treatment-effects estimation using lasso (Telasso) estimator. Telasso estimates the average treatment effect (ATE), the average treatment effect on the treated (ATET), and the potential-outcome means (POMs) using augmented inverse-probability weighting (AIPW) in tandem with estimating lasso for selection of potential control variables to include in the model. Compared with traditional potential-outcome models, it affords the advantage of robust estimation; correctly specify only one of the models and guarantees Neyman orthogonality as lasso guard against model-selection mistakes. In our application, we used the same set of covariates as in lasso regression for outcome model and controlled for a different set of covariates expected to drive job loss in treatment model.

## 3. Result and discussion

### 3.1. Descriptive statistics

Having described the nature of the sample data and the econometric methods applied, we proceed to present the socio-economic characteristics of respondents and results of our analysis in this section. In Table 1, we present the description of our study sample.

**Table 1:** Descriptive statistics of variables used in the analysis.

Variable	Description of variables	Mean	St.Dev
**Food security**			
child_hunger	Child gone hungry in last 7 days (Yes = 1)	0.160	0.366
food_money	Household ran out of money to buy food (Yes = 1)	0.490	0.500
**Employment**			
workingW1	An individual was working in April 2020 (Yes = 1)	0.419	0.493
paidleaveW1	An individual on paid leave in April 2020 (Yes = 1)	0.173	0.378
furloughW1	An individual was furloughed in April 2020 (Yes = 1)	0.118	0.323
Unemployed	Job due to COVID-19 lockdown (Yes = 1)	0.286	0.452
**Social grants**			
no_csgW1	Total child support rant received by household	1.321	1.573
no_oapW1	Total old age pension grant received by household	0.344	0.602
chgrnat_unem	Total child support grant received by household & job loss	0.471	1.130
no_oapW1_unem	Total child old age grant received by household & job loss	0.117	0.388
hh_opg	Living with household receiving old age	0.273	0.445
**Covariates for outcome model**			
w1_female	Female respondent (Yes = 1)	0.555	0.497
rural	Respondent lives in a rural area (Yes = 1)	0.806	0.396
urban	Respondent lives in urban area (Yes = 1)	0.0396	0.195
race1	African (Yes = 1)	0.822	0.381
race2	Coloured (Yes = 1)	0.088	0.283
race3	Asian/Indian (Yes = 1)	0.010	0.103
race4:	White (Yes = 1)	0.046	0.209
hhsizeW1	Household size	5.148	3.153
educ:	Respondent’s education level in year	10.73	2.451
age group 17–24:	age category between 17–24	0.08	0.27
age group 25–34:	age category between 25–34	0.32	0.46
age group 35–44:	age category between 35–44	0.33	0.47
age group 45–54:	age category between 45–54	0.2	0.4
age group 55–64:	age category between 55–64	0.06	0.24
age group 65–102:	age category between 65–102	0.02	0.15
nkids6W1:	number of kids	0.772	1.043
lnhhinc_pcW1:	logarithm of household per capita income	7.626	2.495
Observations		3,408	3,408

Our descriptive analysis that suggests over half of our sample are female, and that roughly over three-quarters of the respondents were Black Africans. Roughly 29% of adults had lost their jobs, whereas 12% were furloughed, since the lockdown came into effect in South Africa on March 27^th^, 2020. About 42% of all adults who were working before the COVID-19 lockdown were still actively working while 17% were on paid leave.

Close to half (49%) of the respondents reported that their household ran out of money to buy food while 16% of the respondents reported that a child went hungry in the last seven days. Average child grant receipts by a household is 1.32 and this estimate drops to 0.471 among households with a member who lost a job due to lockdown. Average receipt of old age pension grant is 0.34 and only 0.117 among households with a member who lost a job due to lockdown. Respectively 7.3% and 2.6% of the respondents were recipients of child support grant and old age pension grant. Average years of education is 10.73, and average household size is 5.148.

### 3.2. Impacts on food insecurity

Having described the nature of the sample data and the econometric methods applied, we proceed to present the results of our analysis in this section. To evaluate the heterogeneity of the effects job losses and thereby evaluate the effectiveness of social grant policies (for child, old age, and COVID-19 grants), we had to estimate the effect of the interaction term on job loss variables with the total receipt of each of these grants. We also added controls for gender, education, household size and location, for example, “living in a metropolitan municipal area during the lockdown” and “race of respondent.” Tables 2 and 3 present detailed estimation results.

**Table 2:** Marginal effect estimates of probit model food security.

Variables	child hunger	household hunger
Unemployed	0.0600***	0.227***
	(2.920)	(5.330)
chgrnat_unem	-0.0182***	-0.0390**
	(-2.594)	(-2.056)
no_oapW1_unem	-0.00163	-0.0869*
	(-0.0853)	(-1.750)
no_csgW1	0.0198***	0.0609***
	(3.654)	(4.907)
no_oapW1	-0.0238	-0.123**
	(-1.073)	(-2.071)
hh_opg	0.0195	0.190**
	(0.589)	(2.497)
Educ	-0.00589***	-0.0143**
	(-2.800)	(-2.309)
hhsizeW1	0.00379	0.00206
	(1.240)	(0.334)
w1_female	0.0364***	0.0215
	(2.915)	(0.707)
urban2	0.00516	0.0334
	(0.371)	(0.815)
urban3	-0.00704	0.105
	(-0.271)	(1.275)
race1	0.0645***	0.193***
	(2.867)	(3.420)
race2	0.0135	0.263***
	(0.292)	(3.655)
race3	-0.000619	0.164
	(-0.0120)	(1.152)
Observations	2,479	3,143

t-statistics in parentheses

*** p<0.01

** p<0.05

* p<0.1

**Table 3:** Lasso (machine learning) marginal effect estimates of food insecurity.

Variables	child hunger	household hunger
unemployed	0.520***	0.590***
	(2.821)	(4.505)
no_csgW1_emp	-0.191***	-0.158***
	(-2.861)	(-2.943)
no_oapW1_emp	-0.133	-0.234*
	(-0.715)	(-1.670)
no_csgW1	0.142***	0.157***
	(2.763)	(4.380)
no_oapW1	-0.171	-0.293*
	(-0.720)	(-1.691)
educ	-0.0857***	-0.0780***
	(-3.928)	(-4.785)
hhsizeW1	0.0969***	0.0143
	(4.591)	(0.906)
w1_female	0.244**	0.157**
	(2.064)	(2.086)
Observations	3,161	3,161

Robust z-statistics in parentheses

*** p<0.01

** p<0.05

* p<0.1

As expected, job loss is a significant cause of food insecurity, regardless of the food insecurity measures considered. In our baseline models (see Table 2), we found that, compared with households housing employed respondents, households with respondents who lost their jobs due to the COVID-19 lockdown were 11.2% more likely to report child hunger and 23% more likely to report household hunger in the past seven days.

Regarding the effects of social grants, we found that receipt of a child support grant by a household with respondents who lost their jobs due to COVID-19 lockdown reduced the likelihood of reporting child hunger and household hunger by 1.8% and 3.9% respectively. The receipt of an old age pension grant, for these households, reduced the likelihood of reporting household hunger by 8.7%with no significant effect on the outcome of child hunger. We also find that, female gender, larger household size, being African race and lower attainment of education of the respondent increase vulnerability to food insecurity

Notice though that the foregoing analysis suffers from model uncertainty, as our selections of variables and the functional form were not adequately informed by economic theory or existing social sciences literature. As mentioned in the previous section, in overcoming this problem we employed the LASSO methods to account for model uncertainty surrounding variable selection. Table 3 presents the estimate of the double-selection logit LASSO models.

As can be seen, many covariates included in the previous model could not survive selection (penalization); therefore, not all the variables in the probit model (Table 2) were selected in the LASSO model. All observations were removed that had a value missing for any one of the variables used in either model, hence the difference in number of observations between the logit model and the LASSO model. Moreover, we see that the estimates of LASSO models are different to those obtained from the traditional probit model, in terms of both magnitude and precision. As such, we consider these estimates to be the correct results for reporting our findings, rather than those presented earlier.

Here again, we find that job loss due to COVID-19 lockdown significantly increased both the child and household hungers with the latter looming larger. To be specific the lasso regression analysis shows that compared with households containing employed respondents, households with respondents who lost their jobs due COVID-19 lockdown were 52% more likely to report child hunger and 59% more likely to report household hunger in the past seven days. However, treatment effect estimates of Telasso, our preferred results, are significantly lower compared with these estimates (see Table 4 below). To be specific, Average Treatment of on the Treated (ATT) estimates suggest that, compared with households containing employed respondents, households with respondents who lost their jobs due COVID-19 lockdown were 5.3%more likely to report child hunger and 2.6% more likely to report household hunger in the past seven days.

**Table 4:** Telasso (machine learning) estimates of food insecurity.

Parameters	child hunger	household hunger
ATE	0.120***	0.0444**
	(4.884)	(2.470)
ATT	0.0539**	0.0256*
	(2.212)	(1.803)
POMs	0.552***	0.139***
	(23.34)	(16.20)
Observations	2,093	3,163

Robust z-statistics in parentheses

*** p<0.01

** p<0.05

* p<0.1

## 4. Discussion

Consistent with what would be expected from studies on unemployment and food insecurity, we find that households with respondents who lost their jobs due to the lockdown were more likely to report child hunger and having run out of money to buy food, suggesting that the lockdown-induced unemployment led to increased food insecurity in South Africa.

Our results are in-line with most findings in the literature [10, 11, 15, 16, 33, 34], apart from [7], who found that despite a significant proportion household having been exposed to job loss or reduced incomes, their food consumption and household dietary diversity remained mostly unchanged or slightly rose by August 2020 in Addis Ababa, Ethiopia. The difference between our result and [10, 11, 15, 16, 33–35] on one hand and [7] on other is attributed to variation in level of lockdown restrictions and their implementation as Ethiopia implemented partial lock down August 2020.

In terms of the magnitudes, our ATT findings are within the range of results of extant literature. A study used a rapid assessment of the impact of COVID19 in May 2020 confirmed that there is little to no detectable rise in food insecurity for all households; a percent of households with children classified as food insecure is about 3% higher than it was in 2016 and 2017 [15]. Moreover, another similar study reported that job loss due to COVID-19 lockdown decreased the likelihood of being food sufficient by 9.5% lower [23]. Our ATT estimates sits within interval of these two findings; 3% and 9.5% rise in food insecurity in response to the lockdown restriction.

However, it is significantly lower than estimates in [11], who found that food insecurity worsened with the proportion of food insecure households rising significantly by 38% and 44% in Kenya and Uganda respectively and estimates of [12], who reported that the lockdown restrictions are associated with 6% to –15% increases in households’ experience of food insecurity in Nigeria From a closer look into these findings, including ours, the following observations can be made. First, the effects of the COVID-19 pandemic on food insecurity are most acutely felt by the least developed countries (LDCs), Kenya, Tanzania and Uganda. Second, variation in estimated effects across the studies are either due to difference in the treatment variables selected, for example, we used job loss as treatment variable, or variation in the level of the lockdown restriction or both across the studies.

Note also that the heterogeneity in the estimated effects, in turn, suggests not only varying adverse short-term welfare implication (decreased individual utility due to food consumption fall), but also heterogenous long-term consequences such as poor health outcomes (particularly among children) resulting from COVID-19 lockdown.

In terms of social grant programs (see Table 3), we found that a receipt of child support grant by a household with respondent who lost jobs due COVID-19 lockdown reduces the likelihood of reporting child hunger and household hunger by 19% and 16% respectively. A receipt of old age pension grant by these households reduces the likelihood of reporting household hunger by 23%with no significant effect on likelihood of child hunger. Note that a closer look into these estimates suggests that the protective effect of social grant across is heterogenous across its alternative programs (child support grant and old age pension grant) and food insecurity outcomes, the results that point to differences in the size of the transfers and motivations for sending these transfers.

Overall, our finding of social grant effects corroborates [26], who confirmed that participation in Ethiopian Productive Safety Net Program (PSNP) reduced the likelihood of food insecurity by 9.3% and food gap by 0.341months.

## 5. Conclusion

In this study, using South Africa’s National Income Dynamics Study (NIDS)—Coronavirus Rapid Mobile Survey (CRAM) data, we investigated the causal effects of job loss and the receipt of social grants on food insecurity during the coronavirus pandemic (April 2020). Unpacking these relationships will help to identify vulnerable populations during the pandemic, inform targeting policy interventions, and shed light on the sufficiency of the current income transfer policies in protecting those vulnerable to labour market shocks and their food insecurity implications.

We find that job losses due to COVID-19 lockdown significantly increased food insecurity in South Africa. To be specific, compared with households housing only employed persons, we find that households with respondents who lost their jobs due to the lockdown were significantly more likely to report child hunger and having run out of money to buy food in the past seven days. We also find that the receipt of social grant programs (child support grant and old age pension grant) shielded people against these effects. Individuals who experienced labour market shock in grant receiving households were less likely to report child hunger and household hunger in past seven days, compared with employed individuals in non-child support grant receiving households. The protective effect of the receipt of an old age pension grant among these households was limited to household hunger. Heterogeneity of the social grant effects across alternative programs (child support grant and old age pension grant) and food insecurity outcomes, suggests the differences in the size of transfers and motivations for sending these transfers.

Our findings have the following important policy implications. First, the significant positive effect of the labour market shock on food insecurity underscores the importance of labour market disengagement as one of channels through which the COVID-19 lockdowns impacted food insecurity with potential adverse health consequences. This highlights the need for income transfer polices to target people that experienced labour market shocks during the pandemic to protect them against food insecurity. Second, the significant negative effect of social grant’s receipt on food insecurity suggests that income transfers are important instruments to close food consumption gaps, especially among those who experienced labour market shocks.

## Supporting information

S1 File (DTA)

S2 File (DO)

## References

[1] Arndt C, Davies R, Gabriel S, Harris L, Makrelov K, Robinson S, et al. Covid-19 lockdowns, income distribution, and food security: An analysis for South Africa. *Glob Food Sec*. 2020; 26: 100410. doi: 10.1016/j.gfs.2020.100410 32834955 PMC7366977

[2] Béné C. Resilience of local food systems and links to food security–A review of some important concepts in the context of COVID‐19 and other shocks. *Food Sec*. 2020;12: 805–822. 10.1007/s12571-020-01076-1 PMC7351643 32837646

[3] Resnick D. (2020). COVID‐19 Lockdowns Threaten Africa’s Vital Informal Urban Food Trade. In *COVID‐19 and Global Food Security*, ed. Swinnen Johan and John McDermott, 73– 4. Washington, DC: International Food Policy Research Institute (IFPRI).

[4] Larborde D, Martin W, Swinnen J, Vos R. COVID-19 risks to global food security. *Science*. 2020: 369 (6503); 500–502. doi: 10.1126/science.abc4765 32732407

[5] Torero M. Without food, there can be no exit from the pandemic. *Nature*. 2020; 580: 588–589. doi: 10.1038/d41586-020-01181-3 32327745

[6] Blustein DL, Duffy R, Ferreira JA, Cohen-Scali V, Cinamon RG, Allan BA. Unemployment in the time of COVID-19: A research agenda. *J Vocat Behav*. 2020; 119: 103436. doi: 10.1016/j.jvb.2020.103436 32390656 PMC7206417

[7] Hirvonen K, de Brauw A, Abate GT. Food Consumption and Food Security during the COVID‐19 Pandemic in Addis Ababa. *American Journal of Agricultural Economics*. 2021; 103 (3): 772–789. doi: 10.1111/ajae.12206 33821007 PMC8013419

[8] WFP. The impact of COVID-19 (Coronavirus) on global poverty: Why Sub-Saharan Africa might be the region hardest hit. 2020a.

[9] WFP. World Bank, Gender Dimensions of the COVID-19 Pandemic. 2020b. The World Bank, Washington, D.C.

[10] Amare M, Abay K, Tiberti L, Chamberlin J. COVID-19 and food security: Panel data evidence from Nigeria. *Food Policy*. 2021; 101. 10.1016/j.foodpol.2021.102099 PMC9758590 36570064

[11] Kansiime MK, Tambo JA, Mugambi I, Bundi M, Kara A, Owuor C. COVID-19 implications on household income and food security in Kenya and Uganda: Findings from a rapid assessment. *World Dev*. 2021; 137: 105199. doi: 10.1016/j.worlddev.2020.105199 32982018 PMC7500897

[12] Devereux S, Béné C, Hoddinott J. Conceptualising COVID-19’s impacts on household food security. *Food Security*. 2020; 12: 1–4. doi: 10.1007/s12571-020-01085-0 32837651 PMC7358330

[13] Galasso E, Ravallion M. Social protection in a crisis: Argentina’s plan Jefes y Jefas. *World Bank Econ Rev*. 2004; 18 (3): 367–399.

[14] Rocco Z. Is employment a panacea for poverty? A mixed-methods investigation of employment decisions in South Africa. *World Development*. 2020; 13, 10493.

[15] Sunjin A, Bailey NF. Measuring food insecurity during the COVID-19 pandemic of spring 2020. *Applied Economic Perspectives and Policy*. 2020; 0: 1–7.

[16] Kijin K, Sunae K, Young P. *Food Security in Asia and the Pacific amid the COVID-19 Pandemic*. Asian Development Bank. 2020. Available from: http://hdl.handle.net/11540/12119. License: CC BY 3.0 IGO.

[17] Casale D, Posel D. Gender & the early effects of the COVID-19 crises in the paid & unpaid economies in South Africa. Working Paper Series NIDS-CRAM Wave 1. 2020.

[18] Ranchhod V, Daniels RC. Labour market dynamics in South Africa in the time of COVID-19: Evidence from wave 1 of the NIDS-CRAM survey.

[19] Mahler DG, Lakner C, Aguilar RAC, Wu H. The impact of COVID-19 (Coronavirus) on global poverty: Why Sub- Saharan Africa might be the region’s hardest hit. 2020a. Available from: https://blogs.worldbank.org/opendata/impact-covid-19-coronavirus-global-poverty-why-sub-saharan-africa-might-be-region-hardest.

[20] Leibbrandt M, Finn A, Woolard I. Describing and decomposing post-apartheid income inequality in South Africa. *Development Southern Africa*. 2012; 29 (1): 19–34.

[21] Rogan M, Skinner C. The COVID-19 crisis and the South African informal economy: ‘Locked out’ of livelihoods and employment. National Income Dynamics Study (NIDS)–Coronavirus Rapid Mobile Survey (CRAM). Wave. 2020 Jul 15; 1:15.

[22] Wills G, van der Berg S, Patel L, Mpeta B. Household resource flows and food poverty during South Africa’s lockdown: Short-term policy implications for three channels of social protection. Stellenbosch, South Africa: Department of Economics, University of Stellenbosch; 2020 Jul 15.

[23] Restrepo BJ, Rabbitt MP, Gregory CA. The effect of unemployment on food spending and adequacy: Evidence from coronavirus-induced firm closures. *Appl Econ Perspect Policy*. 2021; 43: 185–204.

[24] Posel D, Oyenubi A, Kollamparambil U. Job loss and mental health during the COVID-19 lockdown: Evidence from South Africa. *PLOS ONE*. 2021; 16: e0249352. doi: 10.1371/journal.pone.0249352 33784339 PMC8009396

[25] Kerr A, Ardington C, Burger R. Sample design and weighting in the NIDS-CRAM survey. Report B. 2020.

[26] Abay KA, Berhane G, Hoddinott J, Tafere K. COVID-19 and food security in Ethiopia: Do social protection programs protect? *Forthcoming*, *Economic Development and Cultural Change*, 2022.

[27] Bellemare M F., Novak L., Contract farming and food security. *Am*. *J*. *Agric*. *Econ*. 2017; 99(2), 357–378.

[28] Garratt E. Food insecurity in Europe: Who is at risk, and how successful are social benefits in protecting against food insecurity? *Journal of Social Policy*. 2020; 49 (4): 785–809.

[29] Bocquier A, Vieux F, Lioret S, Dubuisson C, Caillavet F, Darmon N. Socio-economic characteristics, living conditions and diet quality are associated with food insecurity in France. *Public Health Nutrition*. 2015; 18 (16): 2952–2961. doi: 10.1017/S1368980014002912 25563304 PMC10271578

[30] Belloni A, Chernozhukov V, Hansen C. Inference on treatment effects after selection among high-dimensional controls. *Rev Econ Stud*. 2014; 81: 608–650.

[31] Belloni A, Chernozhukov V, Hansen C, Kozbur D. Inference in high dimensional panel models with an application to gun control. *J Bus Econ Stat*. 2016; 34: 590–605.

[32] Chernozhukov V, Hansen C, Spindler M. Post-selection and post-regularization inference in linear models with many controls and instruments. *Am Econ Rev*: *Papers & Proceedings* 2015; 105: 486–490.

[33] Gunes F. *Penalized Regression Methods for Linear Models in SAS/STAT*. SAS Institute Inc. 2015.

[34] Ruszczyk HA, Rahman MF, Bracken LJ, Sudha S. Contextualizing the COVID-19 pandemic’s impact on food security in two small cities in Bangladesh. *Environment and Urbanization*. 2020; 33 (1): 239–254. 10.1177/0956247820965156 PMC8261459 34253941

[35] Erokhin V, Gao T. Impacts of COVID-19 on trade and economic aspects of food security: Evidence from 45 developing countries. *Int*. *J*. *Environ*. *Res*. *Public Health*. 2020; 17: 5775. doi: 10.3390/ijerph17165775 32785155 PMC7459461

